# Differences in the processing of anaphoric reference between closely related languages: neurophysiological evidence

**DOI:** 10.1186/1471-2202-9-55

**Published:** 2008-06-27

**Authors:** Monique J Lamers, Bernadette M Jansma, Anke Hammer, Thomas F Münte

**Affiliations:** 1 Department of Linguistics, Radboud University Nijmegen, The Netherlands; 2 Faculty of Psychology, Department of Neurocognition, Maastricht University, Maastricht, The Netherlands; 3 Department of Neuropsychology, Otto-von-Guericke-Universität Magdeburg, Germany

## Abstract

**Background:**

The present study examines the involvement of syntactic and semantic/conceptual processes in the comprehension of pronouns in Dutch using the technique of event-related brain potentials (ERPs) replicating and extending an earlier study in German. Dutch and German are closely related and share the same logic in referring to non-diminutive and diminutive NPs (i.e. adding an affix which changes the syntactic gender into neutral). Both languages separate male (*hij*/*er *(*he*)) and female pronouns (*zij*/*sie *(*she*)), as well as a pronoun that refers to an entity of neutral gender, (*het/es *(*it*)). However, the neutral pronoun *het *in Dutch is not only a pronoun, it also is the article of a neutral noun. To investigate the influence of this word class ambiguity on pronoun resolution, as well as to establish the generality of the finding of the German study we manipulated syntactic and biological gender congruency between a personal pronoun and its antecedent in Dutch.

**Results:**

In Dutch, sentences with the word-class (pronoun/article) ambiguous pronoun *het *elicited an early negative shift (150–280 ms) which continued in the time frame of the N400. For sentences with a syntactically and biologically incongruent pronoun a P600 (in absence of an N400) was obtained, which was independent of the morphological form of the referent.

**Conclusion:**

The neurophysiological pattern found for Dutch stimuli was clearly different from the German study, indicating that the processing of pronouns in these two languages differs. This can be explained in terms of language specific characteristics concerning the word class ambiguous neutral pronoun *het*. Moreover, in contrast to the findings in the German study, there was no clear effect caused by the morphological form of the referent. Additionally, in Dutch, the pronoun resolution in sentences with a non-diminutive antecedent seems to reflect processes of revision (P600 in absence of an N400), whereas for German evidence was found for clear involvement of conceptual/semantic processes as well as structure building processes (N400/P600 complex).

## Background

The main goal this paper is to investigate the role of semantic and syntactic gender information in pronoun resolution. We performed an ERP study in Dutch, patterned after Schmitt (now Jansma), Lamers and Münte [1] who had used German materials, thus addressing the generalisation of pronoun resolution in closely related languages. Personal pronouns play an important role in discourse understanding. They form cohesive links between sentences and sentence fragments by referring back to a linguistic element, the antecedent, in a so-called co-referential relationship. To establish a cohesive link between the pronoun and its antecedent (i.e. the man, the woman, the child) the pronoun inherits the gender (masculine, feminine, or neuter) and number (singular and plural) characteristics of the antecedent. From behavioural psycholinguistic experiments, there is ample evidence that gender as well as number agreement helps to identify the antecedent, and thus facilitates the comprehension process [2-4]. If the antecedent refers to a human being, gender information is twofold. On the one hand gender can reflect semantic (conceptual, biological) gender information on whether a word refers to a male or female person, or it can reflect whether the word is masculine or feminine, but also neuter. We will refer to the latter as syntactic gender information. Usually male persons are referred to by masculine words and female persons by feminine words. There are, however, also languages in which syntactic and semantic gender do not always go hand in hand. In Dutch as well as in German a diminutive NP is formed by adding an affix (the affix *-je *for Dutch and *-lein *or ^-^*chen *for German). By changing the morphological form, the syntactic gender of the noun changes from masculine or feminine into neuter, but the biological gender stays the same. For example, the diminutive form of Dutch *de jongen *(*the boy*) is *het jongetje *(*the little boy*), which represents a person with male biological gender but is of neuter syntactic gender. The syntactically correct pronoun referring to a neuter noun in Dutch is *het *(*it*). Thus, the biologically congruent male pronoun *hij *(*he*) is syntactically incongruent. As was pointed out by Schmitt, Lamers and Münte [1], this characteristic makes it possible to investigate the role of syntactic and semantic gender information. Moreover, the registration of event related brain potentials (ERPs) parallel to the resolution of personal pronouns makes it possible to gain insight in the time course of pronoun resolution and the involved mechanisms [5-7]. Recently, ERP studies on pronoun processing have shown, that a disagreement in (syntactic) gender information between pronoun and antecedent reveals a so-called P600 effect compared to congruent gender information indicating an involvement of syntactic gender information processing [1,8,9]. Other studies, however, have reported N400-effects (without any positivities) as a reflection of biological/conceptual gender disagreement between the pronoun and the antecedent [[10], see also [11]].

Manipulating semantic and syntactic gender information separately, Schmitt, Lamers and Münte [1] also used this method to study the comprehension of German sentences with different personal pronouns (er/he, es/it, sie/she) referring to a non-diminutive or diminutive antecedent representing a person with a clear male or female gender. An example of a set of the sentences used in the German study [1] is given in (1).

(1) a. Der Bub ist ängstlich und darum legt er/es/sie eine Taschenlampe unter das Bett.

b. Das Bübchen ist ängstlich und darum legt er/sie/es eine Taschenlampe unter das Bett.

(*The boy/little boy is afraid and therefore he/it/she puts a flashlight under the bed*.)

Based on the differences in ERP-waveforms, Schmitt and colleagues [1] came to the conclusion that both syntactic (indexed by the P600 component) and semantic/conceptual (indexed by the N400 component) processes are involved in the resolution of personal pronouns. This pattern was found only for the processing of pronouns referring to a non-diminutive antecedent as in (1b). Therefore, it was suggested that establishing a co-referential relation between a pronoun and a diminutive NP might be purely syntactically driven. Furthermore, differences in distribution of the late positive shift indicated that for the conditions in which both semantic and syntactic gender were violated (i.e. *der Bub*_Masculine_/*das Bübchen*_Neuter_...*sie*; Eng: *the boy*_Masculine_/*the little boy*_Neuter_...*she*) a widely distributed P600 was found, whereas for a purely syntactic gender violation (i.e. *das Bübchen*_Neuter_...*er*; Eng: *the little boy*_Neuter_...*he*) a P600 was found on more frontal electrode sites. In addition, the P600 showed a more parietal distribution for the condition with a biological gender violation, i.e. a diminutive antecedent and correct neutral pronoun (e.g., *das Bübchen*_Neuter_...*es*_Neuter_; Eng: *the little boy*_Neuter_...*it*_Neuter_). The question arises whether similar differences will come about in a closely related language, such as Dutch. Although Dutch and German are similar in both diminutive forming and the formal rules on how pronouns inherit gender and number information from their antecedents, there are also some crucial differences. As will be shown below, these differences seem to affect the actual application of the formal syntactic rules of number and gender agreement between diminutives and the neutral pronoun by native speakers. To investigate the impact of these differences on pronoun resolution as well as the generality of the finding of Schmitt and colleagues, we present a study in Dutch with similar manipulations as were used in the German study.

As in the German study of Schmitt, Lamers and Münte [1] a diminutive or non-diminutive NP representing a male or female person was presented as the subject of a main clause and was followed by a personal pronoun (he/she/it) as the subject of a subordinate clause (see Table 1). In (2a) (Table 1) the syntactically correct pronoun *hij *(*he*) is used to refer to *de jongen *(*the boy*). This condition resembles the German example (1a) in which the pronoun is congruent with the non-diminutive noun on both the syntactic (S+) and the biological (B+) level and is therefore the control condition in the experiment (Table 1, 2a; N/S+B+). Note, however, that in Dutch the same common gender determiner *de *(*the*) is used for male and female noun (*de/the*), whereas in German, they are distinct (i.e. *der *for male and *die *for female nouns). If the main clause is followed by a subordinate clause with the pronoun *het *(*it*), the pronoun is incongruent in both syntactic (S-) and biological gender (B?) (Table 1, 2b; N/S+B?). A question mark is used to distinguish this condition from the condition in which a pronoun of the opposite sex is used as in (2c) in Table 1. In sentences with a non-diminutive antecedent and a pronoun of the opposite sex both syntactic and semantic gender are incongruent (Table 1, 2c; N/S-B-, double violation). For sentences with a diminutive antecedent, the question arises whether it is possible to form a correct condition for both the syntactical and biological gender level. Recall that in Dutch, as well as in German, the gender of a diminutive is neuter. Therefore, formally the syntactic gender of a biologically correct pronoun is incorrect (Table 1, 2d; D/S-B+). If a diminutive NP is followed by a neutral pronoun, the biological gender is incorrect (Table 1, 2e; D/S+B?). If the pronoun of the opposite sex is used following a diminutive antecedent, both levels are violated (Table 1, 2f; D/S-B-, double violation).

**Table 1:** Example sentences.

(2)	a.	N/S+B+	**De jongen **kliert graag en daarom legt **hij_mas _**een gummispin in de koektrommel.
	b.	N/S-B?	**De jongen **kliert graag en daarom legt **het_neut _**een gummispin in de koektrommel.
	c.	N/S-B-	**De jongen **kliert graag en daarom legt **zij_fem _**een gummispin in de koektrommel.
	*translation*		***The [masculine] boy ****likes to tease and therefore ****he/she/it ****puts a rubber spider in the biscuit-tin*.
			
	d.	D/S-B+	**Het jongetje **kliert graag en daarom legt **hij_mas _**een gummispin in de koektrommel.
	e.	D/S+B?	**Het jongetje **kliert graag en daarom legt **het_neut _**een gummispin in de koektrommel.
	f.	D/S-B-	**Het jongetje **kliert graag en daarom legt **zij_fem _**een gummispin in de koektrommel.
	*translation*		**The [neuter] little boy **likes to tease and therefore **he/she/it **puts a rubber spider in the biscuit-tin.

Example sentence with a male (biological gender) subject in the main clause taken from the materials used in the experiment. Sentences (1)a-c have a non-diminutive NP as referent, whereas (1)d-f a diminutive.

Notice that both Dutch and German have a pronoun that refers to an entity of neutral gender, (*het/es *(*it*)), as well as separate male (*hij*/*er *(*he*)) and female pronouns (*zij*/*sie *(*she*)). However, the neutral pronoun *het *in Dutch is not only a pronoun, it also is the article of a neutral noun, as in *het boek (the book)*, whereas there is no such word class ambiguity for the corresponding German pronoun *es*. In fact, the referential status of *het *referring to a diminutive representing a person is questionable. Whereas Schmitt, Lamers and Münte [1], reported a frequency count of 37% for sentences with a pronoun *es *referring with a diminutive NPs in comparison to 63% of occurrences of the biologically congruent pronoun (*er/sie*), for Dutch a frequency count in the text corpus of the Institute for Dutch Lexicology (INL) failed to show any occurrences of the pronoun *het *following a diminutive noun. Thus, although a neutral pronoun referring to a diminutive is syntactically congruent, the language user seems to prefer to use the biologically congruent pronoun (i.e. *hij *or *zij*). Nevertheless, according to the Celex frequency count [12], it can be taken that the usage of *het *as a pronoun is comparable to usage of *hij *and *zij *(*het*: pronoun _log.freq _= 3.92; *hij*: pronoun _log.freq _= 4.05; *zij*: pronoun _log.freq _= 3.40). Since we already have seen that *het *is hardly used referring to a diminutive person, in these instances it almost exclusively refers to things or functions as a dummy pronoun. A further characteristic of *het *is that according to Celex its total frequency is higher than that of the other pronouns (het: article _log.freq _= 4.31).

As a consequence of both the frequency difference and the preference to refer to diminutive NPs that represent male or female persons with the masculine and feminine pronouns (respectively), it is most likely that *het *is initially interpreted as an article rather than as a personal pronoun. Moreover, this would imply a strong expectation for the following word to be a noun. This is neither the case for the two other pronouns *hij *and *zij *nor for the neutral pronoun *es *in the German study. To control for the effect of the subcategorisation frame preference to interpret the neutral pronoun *het *(*it*) as an article, all pronouns were followed by the indefinite article *een *(*a/an*), Thus, differences in ERP-waveforms can be expected at and beyond the pronoun between sentences with the pronuon het and the pronouns hij and zij, but also in comparison to the German data of our group [1].

Thus, whereas the use of diminutives in the German study showed us what the underlying mechanisms are in pronoun resolution in case of a conflict between syntactic and conceptual gender agreement, the Dutch study will help us to reveal how these processes are affected by language specific characteristics such as referent status of a word class ambiguous pronoun.

Predictions for the conditions with the male/masculine and female/feminine pronouns *hij *and zij (respectively) are based on the findings in German [1]. As mentioned above, this study revealed either an N400/P00 complex or a P600 with a different distribution in case of a biological/conceptual and syntactic gender violation (see also [8]). In comparison to the congruent sentences (N/S+B+) an enlarged N400 in combination with a P600 is expected for the sentences with a biological gender violation and a non-diminutive pronoun (N/S-B? and N/S-B-) indicating semantic/conceptual problems as well as problems in final structure building, whereas the processing of a syntactically and/or biologically incongruent pronoun following a diminutive NP might only elicit an enhanced late positivity.

## Results

### Comprehension questions

To check whether the subjects were actually reading the sentences each subject answered 12 simple yes/no questions addressing the content of the sentence they immediately followed. On average, subjects answered these questions 90% correctly (worst subject 85%) indicating no problems in understanding the sentences and showing that they actually did read the sentences.

### Event related brain potentials to the pronoun

**Figure 1:**
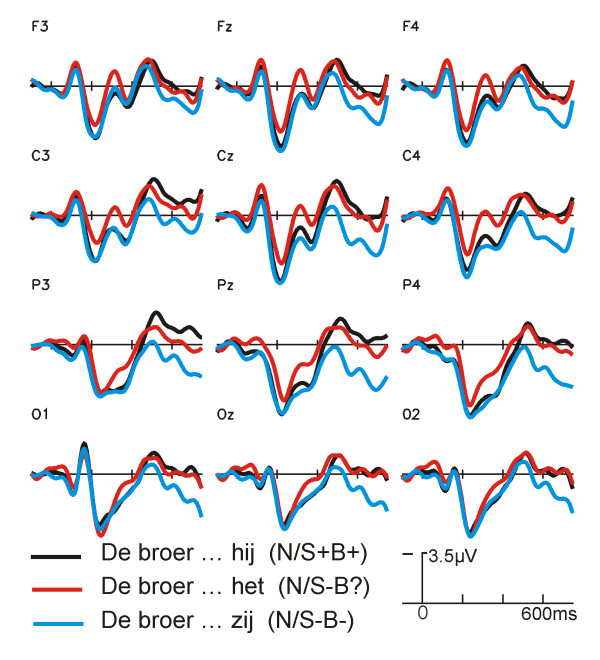
**Grand average ERPs at the pronoun: non-dimutive antecedents**. Grand average ERPs (N = 17) time locked to the onset of the critical pronoun for selected electrode sites for sentences with non-diminutive antecedents. Conditions and their labels are illustrated in Table 1.

The grand average ERPs, time-locked to the onset of the critical pronoun with a baseline 100 ms before the onset of the pronoun, are shown in Figure 1 for the three conditions with a non-diminutive (left panel) and the three conditions with a diminutive referent (middle panel). All 6 conditions elicit a P1, and a N1-P2 complex that is typical for visually presented material.

**Figure 2:**
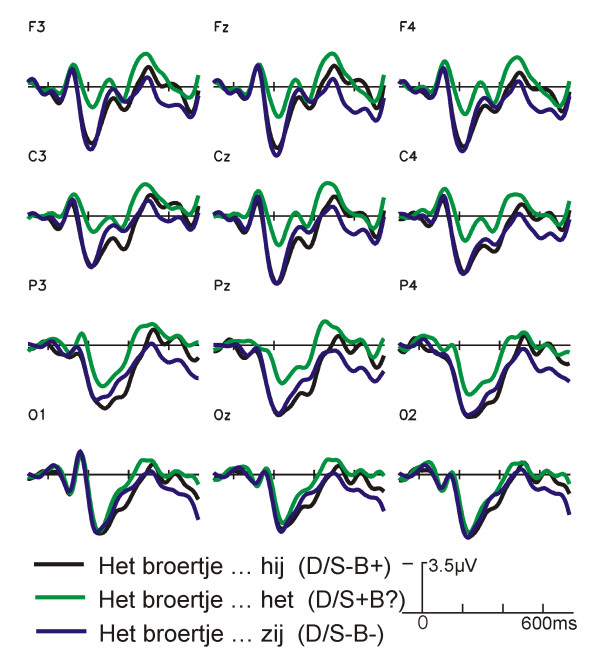
**Grand average ERPs at the pronoun: diminutive antecedents**. Grand average ERPs (N = 17) time locked to the onset of the critical pronoun for selected electrode sites for sentences with diminutive antecedents. Conditions and their labels are illustrated in Table 1.

**Figure 3:**
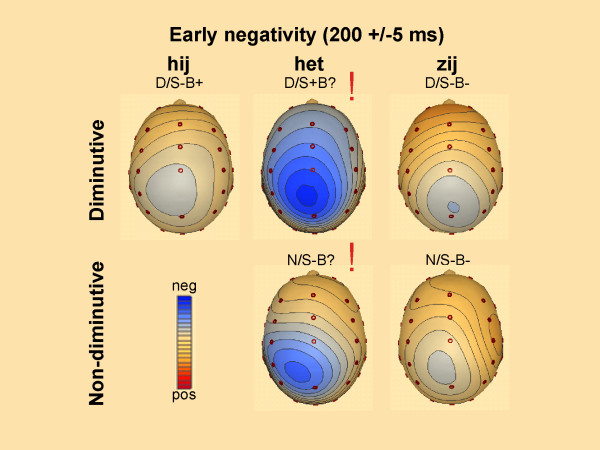
**Spline interpolated maps of the early negativity**. Spline interpolated maps based on the mean amplitude of the difference waves (condition minus baseline (N/S+B+) for the early negativity. Significant effects of condition in the pairwise comparison to the baseline condition are marked with !. Contour lines are presented in steps of 0.10 μV.

An early divergence is visible between ERP waveforms of the conditions with the neutral pronoun (N/S-B? and D/S+B?) and the other conditions. This difference becomes larger between 280 and 400 ms, i.e. for the P2 deflection (Fig. 1, Fig 2). The amplitude of the P2 is smallest for the sentences with a neutral pronoun and a diminutive antecedent (see Fig. 3 for topographical distribution).

Further along, around 300 ms, a negative shift can be noticed in the ERP-waveforms, with (still) a stronger negativity for the sentences with the neutral pronoun compared to the other conditions. This difference is also visible on posterior electrode sites (see Figure 3 for topographical distribution). A small negative shift can be noticed between the diminutive sentences with a biologically congruent but syntactically incongruent pronoun (D/S-B+) and those with a double violation (D/S-B-). Figures 1 and 2 show that there is hardly any difference between sentences with a neutral pronoun and a non-diminutive and diminutive antecedent. The non-diminutive condition with a double violation (N/S-B-) shows a long lasting positivity starting approximately 500 ms after onset of the pronoun in comparison to the two other conditions with a non-diminutive referent (Fig. 2). A similar deviation is visible between the sentences with a double violation and a diminutive referent (D/S-B-) in comparison to the other two conditions with a diminutive referent (Fig. 2).

### Data analysis on the pronoun

The data were statistically evaluated using mean amplitudes computed for each subject and each condition with 100 ms before the onset of the pronoun as the baseline for three different time windows: 150–280 ms for the early negative shift, 280–400 ms for the N400-effect, and 500–800 ms for the late positive shift. To determine the onset and ending of the difference in ERP waveforms the difference between conditions D/S-B+ and D/S+B? in consecutive windows of 12 ms (3 data points) each on Cz was tested. The first window started at the onset of the pronoun; the next window moved 4 ms (1 datapoint) and was therefore overlapping 8 ms (2 data points) with the previous window. To minimize the danger of false positives, an effect was only considered significant when three successive time windows showed these effects (p < .05). The onset of the difference was determined at 150 ms ending at 280 ms. For each window an overall repeated measures of analyses of variance (ANOVA) was carried out. In this ANOVA the mean value of 5 electrode sites in the left anterior (F3, FC3, FT7, F7, FP1), right anterior (F4, FC4, FT8, F8, FP2), left posterior (P3, CP3, TP7 T5, O1), and right posterior (P4, CP4, TP8 T6, O2) quadrant were calculated. This "quadrant" 3 × 2 × 2 × 2 ANOVA included Pronoun (hij/het/zij, representing syntactic and biological gender congruency depending on the gender characteristics of the antecedent), Referent (non-diminutive/diminutive), Hemisphere (left/right position of the quadrants) and Anteriority (anterior/posterior position of the quadrants). In addition, planned pairwise comparisons between conditions were performed.

### Early negative shift (150–280 ms)

In the quadrant ANOVA a main effect of Pronoun was found. There were significant interactions of Pronoun × Hemisphere, Pronoun × Anteriority, and a three way interaction of Pronoun × Hemisphere × Anteriority indicating that there are differences between the sentences depending on Pronoun with differences over the scalp. Notice that no significant effects including Referent were found, indicating that differences in ERP waveforms cannot directly be attributed to differences in the form of the antecedent. ANOVA results are reported in Table 2 for the early negative shift (150–280 ms).

**Table 2:** Quadrant ANOVA at the pronoun.

*Source*	*150–280 ms*	*280–400 ms*	*500–800 ms*
**Pronoun**	17.17**	16.22**	4.92*
**Referent**	xx	xx	xx
**Pronoun × Hemisphere**	4.92*	3.59*	xx
**Pronoun × Anteriority**	8.88**	7.10**	4.97*
**Pronoun × Hem. × Ant.**	4.29*	5.03*	5.03*

Quadrant ANOVA at the pronoun position with factors Pronoun (hij, het, zij), Referent (diminutive, non-diminutive), Hemisphere (Hem.: left, right), Anteriority (Ant.: anterior, posterior) for the 150–280 ms, 280–400 ms and the 500–800 ms.

Note: * p < 0.05; ** p < 0.01 Huynh-Feldt corrected p-values; degrees of freedom for main effect referent (1,16), all other contrasts (2,32)

**Figure 4:**
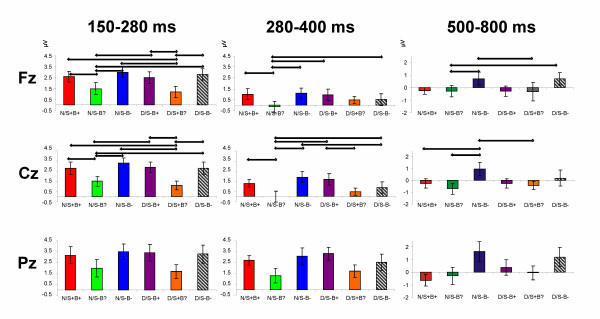
**Bar graphs illustrating mean amplitudes in the pertinent time windows**. The bar graphs depict the mean amplitude in the respective time windows. Also, the results of pair-wise comparisons between the conditions are illustrated by the horizontal bars.

Scalp distribution of difference waveforms (each condition minus the correct condition (N/B+S+)) plotted in Figure 3 revealed a posterior maximum which was confirmed by topographical analysis (i.e. interaction with anteriority, see also Table 2). A further illustration of the mean amplitudes in the respective time-window can be found in Figure 4. Planned pair-wise comparisons showed that in this early time window the waveforms conditions with the ambiguous neutral pronoun *het *(N/S-B? and D/S+B?) differed from almost all other conditions (marked by the exclamation mark in Figure 3, see also Table 2). These negative shifts for the *het *conditions were broadly distributed over the scalp. Notice, however, that between the diminutive condition with the neutral pronoun *het *and the diminutive condition with the double violation (D/S-B? vs D/S-B-) no such difference came about (Table 3).

**Table 3:** Planned pair-wise comparisons at the pronoun.

Early negativity (150–280 ms)					
**Pz**	N/S-B?	N/S-B-	D/S-B+	D/S+B?	D/S-B-
N/S+B+	3.40**	0.72	1.60	2.12*	0.34
N/S-B?		3.60**	4.90**	0.73	3.57**
N/S-B-			0.62	2.20*	1.13
D/S-B+				2.47*	1.69
D/S+B?					1.55

N400 (280–400 ms)					

**Pz**	N/S-B?	N/S-B-	D/S-B+	D/S+B?	D/S-B-
N/S+B+	3.14**	0.72	2.11*	2.11*	-0.34
N/S-B?		3.77**	4.26**	0.73	3.57**
N/S-B-			0.43	2.20*	1.13
D/S-B+				2.86**	1.42
D/S+B?					1.58

P600 (500–800 ms)					

**Pz**	N/S-B?	N/S-B-	D/S-B+	D/S+B?	D/S-B-
N/S+B+	0.53	4.19**	2.18*	1.18	2.79**
N/S-B?		3.73**	0.86	0.41	2.56*
N/S-B-			1.92*	3.42**	0.82
D/S-B+				0.80	1.13
D/S+B?					2.25*

Overview of the T – values of planned pair-wise comparisons between six conditions of the data at the pronoun for three different time-windows (T-values marked with * are significant at the 5% level, and with ** on the 1% level).

### N400 (280–400 ms)

**Figure 5:**
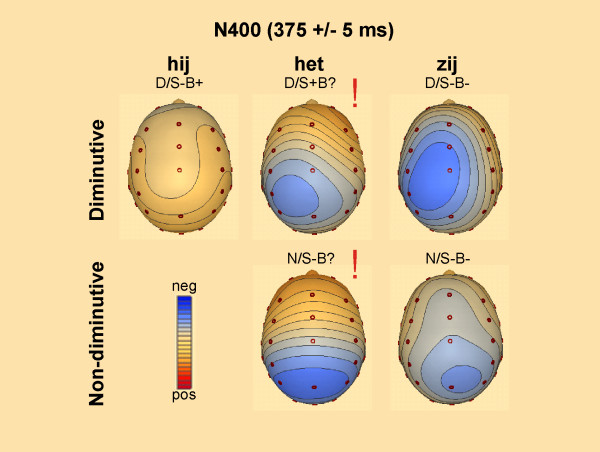
**Spline interpolated maps of the N400**. Spline interpolated maps based on the mean amplitude of the difference waves (condition minus baseline (N/S+B+) for the N400 effect. Significant effects of condition in the pairwise comparison to the baseline condition are marked with !. Contour lines are presented in steps of 0.10 μV.

**Figure 6:**
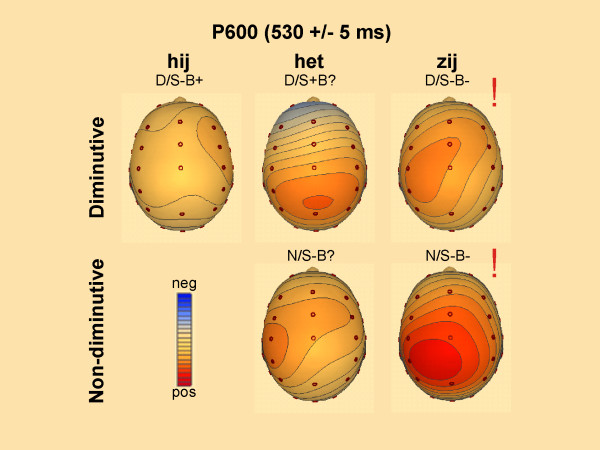
**Spline interpolated maps of the P600**. Spline interpolated maps based on the mean amplitude of the difference waves (condition minus baseline (N/S+B+) for the P600 effect. Significant effects of condition in the pairwise comparison to the baseline condition are marked with !. Contour lines are presented in steps of 0.10 μV.

The second latency window (280–400 ms) was chosen following previous pronoun research [1,8,13]. The quadrant ANOVA for the N400 time range showed a main effect of Pronoun. There were significant interactions of Pronoun × Hemisphere, Pronoun × Anteriority, as well as a three way interaction of Pronoun × Hemisphere × Anteriority (Table 2). In contrast to the German study, no significant effects including Referent were found. As in the previous time window, both conditions with the ambiguous neutral pronoun *het *(N/S-B? and D/S-B?) elicit clear negative posterior deflections compared to all other conditions (Figure 5 and 6 for topographic effects). This difference in waveforms is not visible for the diminutive condition with the neutral pronoun *het *and the diminutive condition with the double violation (D/S-B? vs D/S-B-). Planned pair wise comparisons between conditions for the 260–400 ms time frame confirmed these findings (Table 3). Thus, it might well be that the difference between the enlarged negativities of the conditions with the neutral pronoun *het *are a continuation of the differences found in the previous window.

As in the early time frame, no differences were found between the non-diminutive congruent condition (N/S+/B+) and the conditions with the double violations (N/S-B-, and D/S-B-, respectively). Following the preference of native speakers to refer to a diminutive with a pronoun of the correct biological gender, no difference was expected between the two conditions with the congruent biological gender. However, the slight positive shift visible for the diminutive condition with a congruent pronoun in comparison to the non-diminutive congruent condition turned out to be significant on Pz (N/S+B+ vs. D/S-B+) (Table 3). Since this positive shift continues and has its maximum amplitude in the P600 time frame, we argue that it is the onset of a P600-effect, elicited by the syntactic incongruency.

### P600 (500–800 ms)

Based on visual inspection a later window was determined matching the classical P600 window often reported in the literature. The quadrant ANOVA for the P600-window showed a significant main effect for Pronoun as well as significant interactions of Pronoun × Anteriority, and a three way interaction of Pronoun × Referent × Anteriority (see Table 2). These differences in topography are illustrated in Figure 4 and 6 in which differences in scalp distribution based on different waveforms are plotted.

Planned pair-wise comparisons showed differences between the non-diminutive congruent condition and both conditions with a double violation (N/S+B+ vs. N/S-B- and N/S+B+ vs. D/S-B-) with more positive going waveforms for the conditions with double violation (Table 3). The non-diminutive double violation was also significantly more positive going than the two conditions with the neutral pronoun (N/S-B? vs. N/S-B-, and D/S+B? vs. N/S-B-). A similar pattern was found between the waveform of the diminutive sentences with the double violation and the two waveforms with the neutral pronoun (N/S-B? vs. D/S-B-, and D/S+B? vs. D/S-B-). Both conditions with the neutral pronoun *het *did not differ from the non-diminutive congruent condition and there was no significant difference between the two conditions with the neutral pronoun *het*. As in the previous window, the enlarged positive shift of the diminutive condition with the syntactically incongruent pronoun turned out to be significantly different from the non-diminutive congruent condition (N/S+B+ vs D/S-B+). As will be discussed below, finding a positive shift for conditions with a syntactic inconguency indicates differences in structure building, possibly involving revision and re-evaluation processes.

### ERPs to "een" (the word following the pronoun)

**Figure 7:**
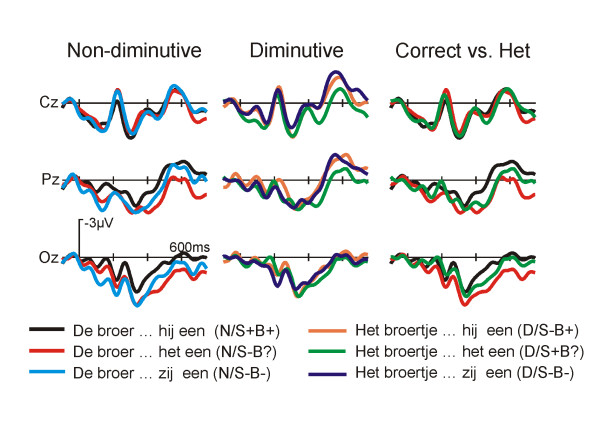
**Grand average ERPs at *een***. Grand average ERPs (N = 17) time locked to the onset of indefinite article *een *the word following the critical pronoun at Fz, Cz, and Pz for sentences with non-diminutive antecedents (left panel), diminutive antecedents (right panel), and a comparison between the two conditions with the neutral pronoun and the correct condition (right panel) Conditions and their labels are illustrated in Table 1.

To investigate the possible subcategorisation frame preference as a consequence of the word class ambiguity of the neutral pronoun *het*, grand average ERPs, time-locked to the onset of *een*, the word following the pronoun, were computed for each condition (Figure 7). Both conditions with neutral pronouns show a positive shift which starts approximately at 350 and lasts until 600 ms in the diminutive condition. For the non-diminutive condition this positivity starts earlier (around 100 ms) but is more prominent from approximately 350 ms onwards. Additionally, a similar positive shift is also seen for sentences with a non-diminutive antecedent and a double violation (N/S-B). Waveforms were quantified by a mean amplitude measure (time window 400–700 ms). The quadrant ANOVA showed a significant main effect for Referent (F(1,16) = 7.79, p < .013) as well as a significant interaction of Referent, Pronoun and Anteriority (F(2,32) = 3.33, p < .048).

Since we suggested that the differences in this time window are related to reanalysis processes caused by the subcategorisation preference introduced by the neutral pronoun, it was expected that planned pair-wise comparisons would reveal differences between the conditions with a neutral pronoun and the other conditions. In Table 4 an overview of the planned pair-wise comparisons on Cz, Pz and Oz are given. As expected, at *een*, the word following the pronoun, the sentences with an ambiguous pronoun (N/S-B?, and D/S+B?) differed from any other condition (N/S+B+, D/S-B+, D/S+B?, and D/S-B-), with the exception of the non-diminutive sentences with the double violation (N/S-B-). In Figure 7 it can be seen that the positive shift for the sentences with a non-diminutive antecedent and the neutral pronoun (N/S-B?) was larger than for the sentences with the diminutive antecedent (D/S+B?). Additionally, the non-diminutive condition with a congruent pronoun was significantly different from the non-diminutive condition with the double violation (N/S+B+ vs N/S-B-). Since the positive shift for the double violation condition already comes about as early as 100 ms after onset of *een *we argue that this positivity is a continuation of the positivity found between these two conditions at the pronoun. This positivity was also significant compared to the diminutive condition with the biologically correct syntactic gender (D/S-B+ vs N/S-B-). Planned pair-wise comparison between the two conditions with double violations did not show a significant difference.

**Table 4:** Planned pair-wise comparisons at *een*.

**Cz** **Pz** **Oz**	N/S-B?	N/S-B-	D/S-B+	D/S+B?	D/S-B-
N/S+B+	-0.27	-0.03	-1.65	0.22	-1.45
	-2.02*	-0.99	-0.35	1.60	0.03
	-3.55**	-1.83*	0.51	2.14*	0.54
N/S-B?		0.35	-1.37	-0.18	-2.35*
		1.30	-2.40*	-1.21	-2.03*
		1.09	-2.63*	-2.64*	-2.64*
N/S-B-			-2.11*	0.31	-2.91*
			-1.36	0.54	-1.38
			-1.57	-0.69	-1.60
D/S-B+				-2.03*	0.85
				-1,99*	-0.18
				-1.19	-0.14
D/S+B?					2.57*
					1.67
					0.99

Overview of the T – values of planned pair-wise comparisons between six conditions of the data at *een *for the 400–700 window on Cz, Pz and Oz (T-values marked with * are significant at the 5% level, and with ** on the 1% level).

## Discussion

The ERP-study presented in this paper assessed the processing of personal pronouns referring to a non-diminutive or a diminutive antecedent in Dutch. We replicated a German study [1] in which syntactic and biological gender congruency of a pronoun was manipulated. The aims of the present study were (1) to investigate the interplay of syntactic and conceptual/semantic information in pronoun resolution in Dutch, and (2) to explore whether the German findings could be generalized cross-linguistically, despite the differences in the pronoun system between German and Dutch. It was pointed out that Dutch and German show similarities in diminutive marking and the pronoun system. However, in Dutch the neutral pronoun *het *can also be an article as in *het mooie boek *(the nice book_neut_). Moreover, from corpus data it can be taken that *het *is hardly ever used in a co-referential relationship with person-antecedents.

The results of the current study in Dutch showed a complex pattern of differences between the ERP waveforms. The ERP-signals time-locked to the critical pronoun showed differences between conditions in three different time-windows at the pronoun: at the 150–280 ms latency window, a window overlapping with the time frame of an N400 (280–400 ms), and a P600 window (500–800 ms). In the first two time frames enlarged negativities were reported for conditions with the neutral pronoun *het *in non-diminutive and diminutive sentences with all other conditions. Crucially, no such difference was found between the diminutive condition with the neutral pronoun *het *and the diminutive condition with the double violation (D/S-B? vs D/S-B-). Additionally, a negativity was found in the 280–400 ms between the diminutive sentences with the correct biological gender and the double violation (D/S-B+ vs D/S-B-). In the later time frame (500–800 ms) late positive shifts were found for conditions with a double violation. Additionally, differences in ERP-waveforms were found at the word *een*, the word following the pronoun in the 400–700 ms window. However, in the German study the ERP waveforms diverged depending on the type of reference. Whereas in German, besides an N400 effect in case of biological gender incongruency between the pronoun and a non-diminutive antecedent and a P600 in case of a syntactic gender incongruency, an N400/P600 complex was revealed if both types of gender were violated. The sentences with the diminutives and an incongruent syntactic and semantic pronoun showed P600-effects without the N400. In Dutch no such dependency on referent type was revealed.

### The involvement of word class ambiguity and frequency differences in pronoun resolution in Dutch

The condition with a neutral pronoun deviated between 150–280 ms clearly from the sentences with a male or female pronoun. Both conditions with a neutral pronoun showed a broadly distributed negative shift in comparison to the other conditions. The two conditions with the neutral pronoun did not differ from each other, notwithstanding the difference in grammatical gender congruency between the two conditions depending on the non-diminutive (S-) or diminutive (S+) antecedent. This indicates that the effect is not caused by differences in antecedent type. Therefore, it seems to be more likely that this early effect is related to word class ambiguity of the pronoun *het *and its preferred interpretation as an article. Federmeier and colleagues [14] compared the ERP-waveforms of word class ambiguous words that were either verbs or nouns with unambiguous verbs and nouns in a sentence context. The word class ambiguous words elicited a frontal negativity with an onset of approximately 150 ms. Hence, the early negativity found in our study might also be caused by the word class ambiguity of *het*. However, the early pronoun effect reported in the current study is more prominent on posterior sites. Investigating differences in processing between open and closed class words, Münte et al. [15] reported a similar more negative going waveform for high frequency closed class words in comparison to medium frequency closed class and open classed words represented in sentences. Based on these findings, we propose that the early negativity found for the neutral pronoun *het *is caused by the word class ambiguity of this pronoun in combination with the difference in frequency.

However, in the 280–400 ms timeframe a positivity was reported between the diminutive biologically congruent and syntactically incongruent pronoun condition and the non-diminutive congruent condition (N/B+S+ vs. D/S-B+). Recall that the biologically congruent pronoun is preferred to refer to a diminutive antecedent. Hence, finding this positivity indicates that, despite the fact that language users regard pronouns that match with the biological gender of the antecedent as correct, the syntactic agreement violation still influences processing.

### The involvement of semantic information in pronoun resolution in Dutch

It was predicted that biological gender congruency would affect the semantic integration process of the pronoun, as reflected in an enhanced N400-effect for the incongruent conditions. However, in Dutch negative shifts in the 280–400 ms time frame were found on central and posterior electrode sites only for sentences with a neutral pronoun *het*. These effects resembled those in the earlier window, suggesting a continuation of the negative shift. No such effects were found for the diminutive biologically congruent condition in comparison to the double violation conditions. Recall that in these conditions there is no unresolved word class ambiguity. We want to point out that the absence of an N400-effect for this condition by no means indicates that the pronoun is fully processed. As will be discussed below, a late positivity was found for these conditions in comparison to the correct condition indicating differences in structure building processes. The absence of an N400 effect indicates, however, that this type of violation is not treated as a problem of semantic integration. An explanation must be sought in the linguistic characteristics of a (personal) pronoun. Because they cannot be used in the same clause as the antecedent they refer to, it might well be that with clear biological and syntactic gender violations between the pronoun and the one available possible antecedent, the parser anticipates for another referent, and therefore no lexical integration problems are encountered at pronoun position (see also [8]).

### The involvement of syntactic information in pronoun resolution in Dutch

It was predicted that sentences with a syntactically incongruent pronoun would elicit a late positive shift, indicating that syntactic information is involved in pronoun resolution. As expected, clear positive shifts were found for the sentences with a double violation in comparison to the correct sentences. These effects are taken as evidence for the involvement of syntactic processing in pronoun resolution. Personal pronouns tend to follow their antecedents with which they form a coreferential relationship. From research on pronoun resolution it is well known that building up a coreferential relationship is well preferred over a disjoint relationship, also known as exophoric pronoun use [4]. We suggest that the P600 reflects the revision that has to take place because of the impossibility to build up the preferred co-referential relationship between an incongruent masculine or feminine pronoun and the antecedent. A similar late parietal positivity was also reported by Matzke et al. [16] for ambiguous sentences that turned out to have a less-preferred non-canonical word order. This positivity was interpreted as indicating revision and re-evaluation processes of the syntactic structure.

In the current study, it is not the structural word order that has to be revised as in the Matzke et al. study, but the preferred binding or bonding between the pronoun and the only possible antecedent. Since in the conditions with the double violations both syntactic and semantic gender information are incongruent and no signs of lexical integration problems, as reflected by the absence of an N400, were found for these sentences, we assume that the P600 is a reflection of problems related to the biological congruency as well as the syntactic congruency (c.f. [1,8,17,18].

Sentences with the neutral pronoun following a non-diminutive antecedent showed no late positive shift, although in this condition the pronoun was incongruent on both gender levels. We argue that the absence of a P600 for these sentences supports the suggestion that *het *is not interpreted as a pronoun, but rather as an article as already discussed above. As expected no positive shift was found between the two conditions in which a syntactically congruent pronoun was used (N/S+B+, D/S+B?), indicating that the resolution of a neutral pronoun following a diminutive NP did not involve any structure building problems.

### Resolving the word class ambiguity

In relation to the word class ambiguity of the neutral pronoun and the preference to interpret *het *as an article, it was expected to find processes of reanalysis one word following the pronoun, the disambiguating indefinite pronoun *een*. This was indeed the case: positive shifts were found at the word *een *following the neutral pronoun *het *in comparison to all other condition, but not in comparison to the condition with the non-diminutive pronoun and the double violation. This indicates that the presence of a syntactically congruent antecedent (i.e. a diminutive NP) helps to resolve the word class ambiguity. Moreover, we argue that this positive shift is a reflection of processes of reanalysis. Following the difference in frequency of the two possible forms of the word class ambiguous word *het*, one can assume that *het *is initially parsed as the more frequently used article and not as a pronoun. At the indefinite article *een *it becomes clear that this preferred interpretation can no longer be maintained. This parsing problem is solved by processes of reanalysis in which *het *has to be reassigned from the article into the less frequently used neutral pronoun [19,20]. Moreover, in a recent study Foraker and McElree [21] argued that in English the resolution of ungendered pronouns (it) takes more time than of gendered pronouns (he, she). They argue that this slow-down reflects the difficulty to recover the intended antecedent. In their study, ungendered pronouns were ambiguous between non-referential use or a referential use referring to an inanimate noun or an event in the immediate discourse. If the wrong interpretation was initially chosen, processes of reanalysis had to occur. If the word following the pronoun provided diagnostic information, revision of the bonding process could take place. In the current study the word *een *following the word class ambiguous pronoun *het *provides such diagnostic information resulting in processes of reanalyses as well as in pronoun resolution. This, however, is not unproblematic since there is no syntactically and biologically congruent antecedent available. Whether the P600 found at the indefinite article following the pronoun reflects processes of disambiguation and/or problems in pronoun resolution can not be determined.

Nonetheless, in the same time frame (400–700 ms) a late positive shift was also found for non-diminutive sentences with a double violation. In this condition the pronoun is incongruent on both the syntactic and biological gender level with only one entity in the sentence that could be the antecedent. Hence, no possible referent is available and the bonding process of the pronoun with an antecedent remains unresolved. We suggest that the positive shift found for the non-diminutive sentences with a double violation is a reflection of this unresolved process, which might entail the ongoing search for a suitable referent. This corroborates the findings of Garrod and Sanford [3], who argue that pronoun resolution is slowed down if antecedent identification is problematic [8,22]. If this reasoning holds, the question arises why no positivity is found for the diminutive sentences with a double violation. As for the non-diminutive condition the pronoun resolution is still unresolved. However, in case of a diminutive antecedent building up a coreferential relationship comes about either with a mismatch between the pronoun and the antecedent of biological gender (i.e. neutral pronoun *het*) or syntactic gender. In this latter case there is biological gender agreement between the antecedent and the pronoun. For building up a coreferential relationship between a non-diminutive antecedent and a pronoun, both syntactic and biological gender need to be matched (as in the N/S+B+ condition). Hence, to establish a disjoint relationship two sources of gender information need to be checked, whereas for diminutive antecedents only one source of information suffices. Therefore, it might be the case that at *een *this issue is already resolved for conditions with diminutive antecedents but not for conditions with non-diminutive antecedents.

### Differences between Dutch and German pronoun resolution

As in the German study of Schmitt et al. [1] we conclude that both syntactic and semantic information was used in the resolution of Dutch congruent and incongruent pronouns. The clear pattern of P600 and N400 effects found in the German study was not replicated in the Dutch study, however. In German, sentences with incongruent pronouns and non-diminutive antecedent showed an N400/P600 complex, whereas diminutive sentences showed an increase of the P600 amplitude in absence of an N400. In the Dutch study, however, language specific characteristics of the neutral pronoun *het *caused a different pattern of effects in the ERP-waveforms. In addition to the N400 effects and the late positive shifts an early negativity was found for the sentences with the neutral pronoun in comparison to the sentences with the masculine and feminine pronouns. We argued that this negativity was caused by word class ambiguity of *het*, which is neither the case for the other pronouns, nor for the pronouns used in the German study. This difference in word class ambiguity between the two languages, however, does not explain why different effects are found in the German and the Dutch study for the sentences with the double violation and a non-diminutive antecedent. In contrast to German, the Dutch non-diminutive sentences with a double violation showed a late positive shift in absence of any N400 effect. We suggested that in Dutch pronoun resolution of incongruent masculine or feminine pronouns involves processes of syntactic structure revision, whereas in German there is also clear involvement of conceptual/semantic processes for non-diminutive antecedents, but not if the only possible antecedent is a diminutive. Moreover, in Dutch no such antecedent dependency was found. This indicates a clear difference in the influence of morphological marking of diminutives between Dutch and German. In comparison to Dutch, German is a language with a richer morphological marking. As a result, morphological marking of the diminutive seems to initiate different processes for pronoun resolution in sentences with non-diminutives and diminutive antecedents. In the absence of the morphological marking, the biological and syntactic mismatch information results in semantic processing difficulties as well as revision processes. In Dutch, however, gender mismatches seem to open the possibility to revise the structure from a preferred structure with a coreferential relationship to a less-preferred structure with an antecedent outside of the sentence, without differences in pure/clear/distinct semantic processing costs.

## Conclusion

In conclusion, differences in ERP-pattern indicate that there are cross-linguistic differences in pronoun resolution between Dutch and German. Part of these differences can be explained in terms of language specific characteristics concerning the word class ambiguity of the neutral pronoun *het*, eliciting a negative shift. Positive shifts at the word following the neutral pronoun underpin that the effects at the neutral pronoun should be attributed to the word class ambiguity and the preferred initial interpretation of *het *as an article and not as a pronoun. Furthermore, in Dutch, the pronoun resolution in sentences with a non-diminutive antecedent seem to reflect processes of revision (P600 in absence of an N400), whereas for German evidence was found for clear involvement of conceptual/semantic processes as well as structure building processes.

## Methods

### Subjects

Seventeen neurologically healthy, right-handed, native speakers of Dutch (age range: 19–28; 12 women) were recruited from the student population at the University of Maastricht and paid for their participation in a single session. Too many artefacts necessitated the rejection of one additional subject.

### Material

Sixty nouns specifying persons' professions, titles or states were selected. Half of these nouns represented clearly male, the other half clearly female persons. These nouns in their diminutive or non-diminutive form served as the subject in sentences consisting of a main clause and a subordinate clause. The subject of the subordinate clause was a pronoun that most likely refers to the subject of the main clause, since no other possible antecedent is available. As described above, the pronoun was either compatible with the antecedent in terms of syntactic and biological gender (S+B+), incongruent on the syntactic level (S-), incongruent on the biological level using the neuter pronoun (B?) or incongruent on the biological level using the pronoun of the opposite gender (B-). Each noun appeared in the diminutive (D) and non-diminutive (N) form, forming six conditions (see Table 1, 1a–1f). As in Schmitt et al.[1] only verbs were used in the subordinate clause that do not allow for a dummy pronoun *het *(*it*) as in *en daarom gaat het regenen (and therefore it will rain*). The used sentences, presented in isolation and using the syntactic construction as just described, minimized ambiguity for co-reference. The experimental sentences ranged from 10 to 14 words in length.

Three different contexts were created for each noun. This made it possible to present each noun three times in a list without repeating complete sentences. The material was distributed across three different lists, counterbalancing the order referent types, pronoun types, repetition, and sentence context. Within a given list, a non-diminutive was presented three times in three different contexts and with three different pronouns. The same held for diminutives, resulting in six main conditions as listed in Table 1. Sentences on a list were pseudo-randomized over six blocks in such a way that each condition appeared 10 times in each block and repetitions of referents (3 times, one for each pronoun) and contexts (2 times, one for diminutives, one for non-diminutives) were kept as far apart as possible.

Sixty filler sentences were added to each block. The filler sentences were the same over the lists. They were included in order to avoid the development of expectancies of sentence continuations. To check whether the subjects were actually reading the sentences, 10 experimental sentences and 2 filler sentences occurring at random positions within a block were followed by a simple yes/no question addressing the content of the sentence. This task was independent of the pronoun manipulation to avoid interference of the explicit comprehension task with the implicit ERP measures of the pronoun manipulations.

Each participant read one list (60 sentences per condition). Participants were equally divided over lists with 6 subjects assigned to each list. Subjects were pooled again for the analysis, except for one subject, which was excluded from the experiment due to too many artefacts in the data set.

### Procedure

Six blocks of 60 experimental sentences (i.e. 10 sentences of each condition) and 60 filler sentences were visually presented in a word-by-word fashion (350 ms word presentation, 300 ms blank screen) in the middle of a video-screen. The words were presented in 16 points font size. After the last word of a sentence a blank screen was shown for 600 ms, followed by a fixation asterisk (1750 ms on the screen, 300 ms blank screen). Each block started with the command *Let op de eerste zin begint nu*. (*Attention, the first sentence starts now*.). In the middle of each block there was a pause of 30 seconds followed by a filler sentence to warn the subject to be prepared for the second part of that block. Each block lasted approximately 25 minutes. The entire experiment, including electrode application and removal took 3.5 hours.

Subjects were tested individually in a dimly lit sound attenuating room facing a colour video screen at a distance of 110 cm. They were instructed to move as little as possible and to read the sentences for content. They were allowed to blink in between sentences as soon as an asterisk appeared on the screen and while answering a question. Subjects had to answer the questions by pressing one of the two mouse buttons. After each block the subjects received feedback on their performance on the questions.

### Data Acquisition and Analysis

Continuous EEG was recorded from 29 scalp sites including all standard sites of the international 10/20 system, using tin electrodes in an electro-cap. Biosignals were rereferenced off-line to the algebraic mean of the activity at the two mastoid processes. Bipolar EOG was recorded between electrodes at the outer left and right canthus and above the eyebrow and below the left eye. The impedance of the electrodes was kept under 5 kOhm. The signals of each electrode were amplified, bandpass-filtered between .05 and 30 Hz, and digitised with a sampling frequency of 250 Hz. The baseline of the waveforms was adjusted on the basis of the averaged activity 100 ms preceding the pronoun onset.

For each of the six conditions average ERPs were computed for each subject with an epoch length of 1024 ms starting 100 ms before the onset of the pronoun. Due to eye movements, blinks, and electrode drift approximately 28% (no differences in conditions) of the trials were rejected.

## Abbreviations

B: biological gender; D: Diminutive; N: non-diminutive; S: syntactic gender; +:congruent; -:incongruent; ERP: event-related potential; NP: noun phrase

## Authors' contributions

MJAL conceived of the study, participated in the design, carried out the data collection, EEG analysis and statistical analysis, interpreted the data and drafted the manuscript, BMJ conceived of the study, participated in the design and the coordination of the study, AH helped with the EEG analysis and statistical analysis, and helped to draft the manuscript. TFM participated in the design, interpreted the data, and helped to draft the manuscript. All authors read and approved the final manuscript.
